# Dose-Response Relationship Between External Load Variables, Body Composition, and Fitness Variables in Professional Soccer Players

**DOI:** 10.3389/fphys.2019.00443

**Published:** 2019-04-17

**Authors:** Filipe Manuel Clemente, Pantelis Theodoros Nikolaidis, Thomas Rosemann, Beat Knechtle

**Affiliations:** ^1^Polytechnic Institute of Viana do Castelo, School of Sport and Leisure, Melgaço, Portugal; ^2^Instituto de Telecomunicações, Delegação da Covilhã, Covilhã, Portugal; ^3^Exercise Physiology Laboratory, Nikaia, Greece; ^4^Institute of Primary Care, University of Zurich, Zurich, Switzerland; ^5^Medbase St. Gallen Am Vadianplatz, St. Gallen, Switzerland

**Keywords:** dose-response, external load, body fat, VO_2max_, isokinetic, association football

## Abstract

The aim of this study was to test associations between accumulated external load and changes in body composition, isokinetic strength, and aerobic capacity of soccer players. Twenty-three professional soccer players (age: 24.7 ± 2.8 years; height: 179.2 ± 6.3; experience: 9.7 ± 2.1 years) participated in this study. This pre-post study design was performed during 10 weeks from July to August of 2017 (4 weeks of pre-season and 6 weeks during the early season). Players were monitored daily by GPS technology and were assessed before and after a 10-week period in terms of body mass (BM), fat mass, lean mass, isokinetic strength at 60°/s, VO_2max_, and HR_max_. Large-to-very large positive correlations were found between the sum of sprinting distance and % differences of BM [0.70, (−0.09;0.95)], HR_max_ [0.51, (−0.37;0.91)], agonist (quadriceps)/antagonist (hamstrings) left ratio [0.84, (0.27;0.97)] and agonist/antagonist right ratio [0.92, (0.58;0.99)]. Large positive correlations were found between the acceleration sum and % differences of VO_2max_ [0.58, (−0.29;0.92)], quadriceps left peak torque [0.66, (−0.16;0.94)], hamstrings left peak torque [0.68, (−0.13;0.94)] and hamstrings right peak torque [0.62, (−0.22;0.93)]. Sprinting load was largely and positively associated with changes in knee strength asymmetries. Acceleration sum was largely and positively correlated with variations at VO_2max_ and peak torques at hamstrings. In addition, dose-response relationships using external load variables were identified in professional soccer players.

## Introduction

Training loads imposed on professional soccer players throughout the season are intended to properly stimulate and improve players’ physical fitness (Impellizzeri et al., 2005). It is expected that chronic exposure to individualized training loads (volume and intensity) contributes to benefits in players’ fitness levels, especially if the training process is adjusted to the needs of individual athletes (Manzi et al., 2009). However, in team sports, and specifically in soccer, the training process extensively uses group-exercises in which the variation of load impact on players can be high (Arcos et al., 2014; Gil-Rey et al., 2015). For that reason, the contributions of generalized training (or specific exercises that do not consider the individualization principle) in soccer players’ performance may not be as high as expected, based on the ranging fitness status of players (Impellizzeri et al., 2005).

The association between training load and the physical and physiological changes in response to training load can be called the dose-response relationship and can be considered one of the determinant components of the training process (Fitzpatrick et al., 2018). The dose-response relationship may differ based on the trainability status of players, the suitability of training stimuli, the stage of the season, or even how well exercises are tailored to each player (Owen et al., 2012). Moreover, the specific method used to determine the load can influence the dose-response relationship (Akubat et al., 2012). Generally, training load can be classified as internal and external (Halson, 2014). Internal load describes the physiological effects of the training stimulus on the psychophysiological variables (e.g., heart rate, perceived exertion) (Campos-Vazquez et al., 2017), whereas external load represents the physical impacts of training (e.g., distance covered at different speed thresholds, acceleration profiles, etc.) (Castagna et al., 2017).

The dose-response relationship has been analyzed using different methods of load quantification as well as different physical and physiological variables (Arcos et al., 2015; Gil-Rey et al., 2015; Fitzpatrick et al., 2018). In terms of internal load, a study conducted among young soccer players revealed large negative associations between accumulated perceived load in the leg muscles and differences in counter-movement jump, and between running velocity and lactate production (Arcos et al., 2015). Accumulated perceived exertion has also been shown to have large relationships with improvement in the highest speed achieved during the 30–15 Intermittent Fitness Test by professional players (Campos-Vazquez et al., 2017). By measuring heart rate (training impulse), large correlations between weekly load and percentage of change in velocity at 2 mmol/L of blood lactate concentration were observed (Akubat et al., 2012). Also using training impulse, large correlations with changes in the maximal rate of oxygen consumption, running speed at 4 mmol/L of blood lactate concentrations, and performance in Yo-Yo Intermittent recovery test level 1 have been recorded (Manzi et al., 2013).

While dose-response studies using external load variables are fewer than those conducted with internal variables (Fitzpatrick et al., 2018), researchers who have studied external load variables have reported important findings. For example, a study conducted on ten amateur players proposed that there is a relationship between external load measures (e.g., player load, mean metabolic power, high intensity distance) and variations in fitness variables (e.g., velocity at lactate threshold, velocity at onset of blood lactate accumulation) (Akubat et al., 2018). The main conclusions of this study revealed large relationships between external load variables and measures of fitness when used as a ratio (Akubat et al., 2018). In a different study conducted on twelve professional players, it was observed that individualized speed thresholds demonstrated a strong dose-response relationship with variations in aerobic fitness (Fitzpatrick et al., 2018).

As mentioned, there are few studies conducted among soccer players which have analyzed the dose-response relationship using external load variables (Akubat et al., 2018; Fitzpatrick et al., 2018). Moreover, the main dose-response relationship investigated the effect in measures of lower-limb (e.g., jumping performance) and aerobic fitness (e.g., distance covered at incremental field tests or estimated VO_2max_). There is no information, to the best of our knowledge, about the dose-response relationship regarding body composition parameters or isokinetic strength. Considering that training load may cause different adaptations in players, it is important to determine the magnitude of such changes and their associations with the physical stimuli imposed by coaches during training. In fact, the knowledge about the changes in fitness levels after a specific training period is relatively well-known for soccer players (Anderson et al., 2016; Los Arcos and Martins, 2018). However, the relationship between those changes and the accumulated load is not well established. For that reason, it is important to understand the association levels between accumulated load during training sessions and the changes in fitness status, while aiming to understand which variations should be done in the training stimulus to optimize the player’s adaptations. Moreover, the implications of associating the load with possible knee strength asymmetries (as an example) can be important to properly managing the load and to avoid extreme deviations in the force production between agonists and antagonists.

Based on the above mentioned reasons, the purpose of this study was to analyze the associations between accumulated external load variables and changes in body composition, isokinetic strength, and aerobic capacity in professional soccer players over a 10-week training period.

## Materials and Methods

### Ethics Statement

The study was approved by the local ethical committee (Polytechnic Institute of Viana do Castelo, School of Sport and Leisure) with the code number IPVC-ESDL180801 and followed the ethical recommendations for the study of humans as suggested by the Declaration of Helsinki.

### Participants

Twenty-three professional soccer players belonging to the same team in the second league of Portugal participated in this study (Table 1). The study included 4 lateral defenders, 4 central defenders, 9 midfielders, 3 wingers, and 3 forward. Goalkeepers were not included in the study. None of the participants had a long-term injury (>4 weeks) during the study and all participants began the study with no injuries. Players were not involved in any other training programs aside from the training regimen imposed by the coach. Players were informed about the research design and the potential risks and benefits of participation, after which, they signed an informed consent. The sample size was tested using the assumption of an alpha of 0.05 and a beta of 0.80. The variable with highest variability coefficient was used to test the sample size. The procedures were made in the GPower 3.17 software. The analysis suggested a minimum sample of 19 players.

**Table 1:** Descriptive statistics of the players’ characteristics at the beginning of the study.

	Age (years old) Mean *(SD)*	Height (cm) Mean *(SD)*	Body mass (kg) Mean *(SD)*	Experience (years) Mean *(SD)*
Players (*N* = 23)	24.7(2.8)	179.2(6.3)	76.75(5.56)	9.7(2.1)

### Experimental Design

A pre-post study design was performed during 10 weeks from July to August of 2017 (4 weeks of pre-season and 6 weeks during the early season). Players were monitored over 47 training sessions and 12 matches during the study. Before and after the 10-week period, the participants were tested to determine their body composition and physical fitness performance. The first assessment took place before players had undergone any high-load training; the second evaluation occurred 48 h after the previous training session. The tests for each period (pre and post-training intervention) occurred on two consecutive days. On the first day, the body composition was assessed during the early morning period, followed by the isokinetic strength tests. On the second day, the aerobic capacity was measured. Players trained 4–8 times a week during the pre-season and 3–5 times a week during the early season. Players were monitored using a validated and reliable 10-Hz GPS unit with accelerometer, gyroscope and magnetometer (100 Hz, 3 axes) during all training sessions.

### Body Composition

Measurements were taken before breakfast (7:30–8:30 a.m.). The players did not take any medications or pharmacological agents that could have affected the assessments in the 24 h before testing. The test assessments were conducted in a room with a stable temperature (23°C) and the procedures of data collection were performed by the same researcher. Body mass (BM), fat mass (FM), and lean mass (LM) were assessed by the bioelectrical impedance method (SECA, mBCA 515, Hamburg, Germany). Body height was assessed with a stadiometer (SECA 242, Hamburg, Germany).

### Aerobic Fitness

After a standardized warm-up protocol consisting of low-intensity running (7 km/h on a treadmill), players performed an incremental test with an initial speed of 8.0 km/h, increasing 0.5 km/h every 30 s until exhaustion (Technogym, Exite Run 600, Italy). Exhaustion was declared voluntarily by the participant or by the incapacity to perform the effort at the predetermined speed. The treadmill slope was fixed at 2%. Values of VO_2max_ were determined using a gas analyzer (Fitmate Pro, Cosmed, Italy) which allowed data to be collected on a breath-by-breath basis. The highest VO_2_ value obtained during each stage was recorded as VO_2max_ after achieving VO_2_ plateau considering the <2 SD (Taylor et al., 1955).

Heart rate (HR) was also assessed during the protocol using a heart rate monitor (H10, Polar, Finland) allowing the researchers to determine the maximal HR (HR_max_) during the effort. The test occurred at the same hour and day of the week for both assessments (pre- and post-10 weeks). The treadmill test was carried out in a room with a stable temperature (21°C) and relative humidity (55%).

### Isokinetic Strength

After a 5-min cycloergometer (Monark LC4, Sweden) warm-up at moderate intensity and mobility exercises focused on lower-limbs, isokinetic strength tests were performed using a Biodex isokinetic dynamometer (System 4 Pro^TM^, United States). The quadriceps (QUADS) and hamstrings (HAMS) torques were gravity corrected and the dynamometer calibration was made accordingly with the manufacturer’s instructions. Both legs were evaluated in a random order after verbal and visual instruction and feedback. After familiarization with the isokinetic movement (non-recorded two trials for each player), the players were tested over five repetitions of concentric knee extensions and flexions at 60°/s. The time of recovery between each repetition was 30 s. Isokinetic strength ratios were calculated from measurements of the maximal QUADS and HAMS peak torques. The test was performed in a room with a stable temperature (21°C) and relative humidity (55%). The following variables were determined: peak torque left anterior (quadriceps) (PTLQ); peak torque right anterior (quadriceps) (PTRQ); peak torque left posterior (hamstrings) (PTLP); peak torque right posterior (hamstrings) (PTRP); and quadriceps/hamstrings ratio (QUADS/HAMS).

### Training Load

Each day, players were tracked with a geolocation tracker (JOHAN Sports, Noordwijk, Netherlands), consisting of a GPS sensor (10 Hz, including EGNOS correction), accelerometer, gyroscope and magnetometer [100 Hz, 3 axes, (16 g)]. The devices are reliable and valid for measuring external load (Nikolaidis et al., 2018). Each player used the same GPS throughout the training period to minimize the inter-equipment variability.

Players first familiarized themselves with the GPS trackers before beginning the experiments. Players wore a body tight vest, and a motion tracker was positioned in a pocket of the vest located in the dorsal region. Motion data were exported daily and treated in the JOHAN sports web analytics platform.

Four load variables were collected during each session: (a) duration of training session (in minutes); (b) total distance (m); (c) sprinting distance at >20.0 km/h (m); and (d) acceleration sum – load (A.U.). The acceleration sum was assessed with the support of the accelerometer. This variable represents the accumulation of data collected from all axes (anteroposterior, mediolateral, and craniocaudal) (Clemente et al., 2017). The sum of training load variables during the 10-week period was calculated to test the associations between the percentage of differences occurring in body composition and fitness variables.

### Statistical Procedures

Results were presented in form of text, tables, and figures, as either means with standard deviation (SD), means with a 90% confidence interval (90% CI) (Batterham and Hopkins, 2006) or coefficient of variation (CV) where specified. The percentage of difference between pre- and post-test outcomes was based on the difference in means (pre-post) multiplied by 100 and divided by the pre-test mean. Normality of the data was tested with Shapiro-Wilk. The data was normal with a *p*-value >0.05 for all the variables. Within-group changes regarding body composition and fitness variables were analyzed using standardized differences of effect size (ES) with a 90% CI (Cohen, 1988). The 90% level seems to be a suitable default, because the chances that the true value lies below the lower limit or above the upper limit are both 5%, that can be interpreted as very unlikely (Batterham and Hopkins, 2006). The following interpretation of ES was used (Hopkins, 2002): <0.2 = trivial; 0.2–0.6 = small; 0.6–1.2 = moderate; >1.2 = large. Probabilities were calculated considering the smallest worthwhile changes (SWC, 0.2 × between-subjects SD) (Hopkins et al., 2009). Qualitative mechanistic probabilistic inferences about the true effects were made using these probabilities (Hopkins et al., 2009). The scale for qualitative probabilities was as follows: 25–75% = possible; 75–95% = likely; 95–99% = very likely; >99% = almost certain (Hopkins et al., 2009). Pearson’s product-moment correlation coefficients tested the relationships between the 10-week sum of training load variables and the % of differences (pre-post) in body composition and fitness variables. The magnitude of correlation [*r*,(90% CI)] between variables were assessed with the following thresholds: <0.1, trivial; 0.1–0.3, small; 0.3–0.5, moderate; 0.5–0.7, large; 0.7–0.9, very large; and >0.9, nearly perfect (Hopkins et al., 2009).

## Results

**FIGURE 1:**
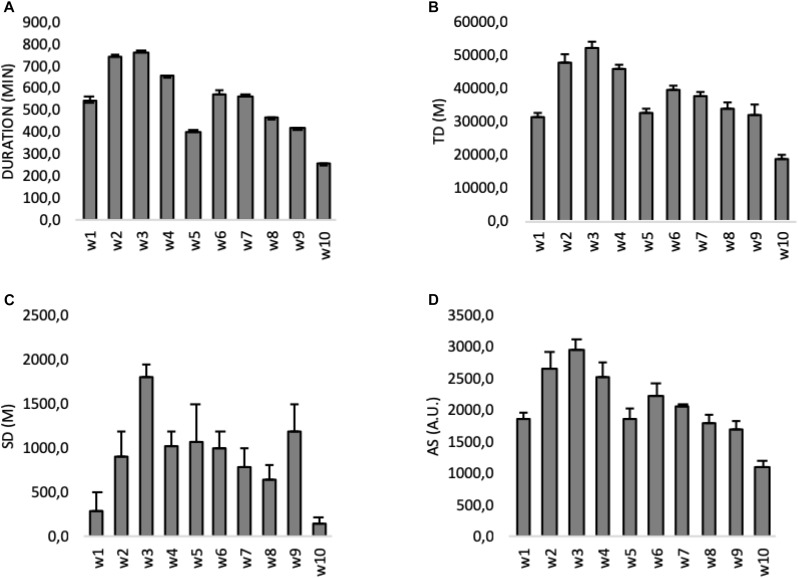
Between-week (w) variations of the sum of load variables for all players. Data are means (±SD). **(A)** weekly sum of duration; **(B)** weekly sum of total distance (TD); **(C)** weekly sum of sprinting distance (SD); and **(D)** weekly sum of accelerations (AS).

The sum of training load variables during the 10 weeks can be observed in Table 2. Variations of load variables during the 10-week period can be found in Figure 1. Duration [90%CI: 446.6;630.1] had a CV of 29.4%. Total distance [90%CI: 31526.01;42717.09] had a CV of 26.0%. Sprinting distance [90%CI: 618.43;1158.01] had a CV of 52.40%. Acceleration sum [90%CI: 1773.51;2388.05] had a CV of 25.47%.

**Table 2:** Sum of training load variables during the 10-weeks.

Duration (min)	TD (m)	*SD* (m)	PL (A.U.)
5383.3(158.3)	371215.5(9652.8)	8882.2(465.4)	20807.7(530.0)

*Data are means (±SD).*

Comparisons were executed between the assessments pre- and post-10 weeks of training, and can be found in Table 3. The main evidence revealed large decreases of LM between pre and post-assessments to be likely [−2.2%, (− 4.7;0.3); ES: −1.44, (−3.08;0.20)], large increases of VO_2max_ to be almost certain [7.5%, (5.2;9.9); ES: 1.92, (1.34;2.50)], large increases of posterior (HAMS) right peak torque as almost certain [11.7%, (6.4;17.3), ES: 1.27, (0.71;1.82)] and large increases of QUADS/HAMS right ratio as likely [10.6%, (1.0;21.1), ES: 1.94, (0.19;3.69)].

**Table 3:** Within-group differences of body composition and fitness variables between pre- and post-10 week period of training.

Variable	M*(SD)*	M*(SD)*	% difference (Post-Pre)		Standardized difference (Post-Pre)		% greater/similar/lower values for Post vs. Pre
	Pre	Post	Value	[90%CI]	Value (*Magnitude*)	90%CI	
BM (kg)	76.75(5.56)	76.78(5.17)	0.1	[−1.1;1.2]	0.01 *trivial*	[−0.13;0.14]	2/97/1 *Very likely*
FM (%)	14.04(1.12)	14.45(1.27)	2.9	[−1.1;7.2]	0.30 *small*	[−0.12;0.73]	68/29/3 *Possibly*
LM(%)	85.96(1.12)	84.10(3.08)	−2.2	[−4.7;0.3]	−**1.44 *large***	[−3.08;0.20]	5/4/91 *Likely*
VO_2max_ (ml/kg/min)	53.38(1.69)	57.40(1.72)	7.5	[5.2;9.9]	**1.92 *large***	[1.34;2.50]	100/0/0 *Almost certain*
HR_max_ (bpm)	183.73(3.35)	180.40(7.36)	−1.9	[−4.2;0.6]	−0.87 *moderate*	[−2.01;0.26]	6/8/86 *Unclear*
PTLA (Nm)	248.01(30.63)	268.53(44.09)	7.8	[0.0;16.2]	0.51 *small*	[0.0;1.02]	86/12/2 *Likely*
PTRA (Nm)	249.59(22.65)	264.90(39.85)	5.5	[−0.5;11.9]	0.50 *small*	[−0.05;1.05]	84/14/3 *Likely*
PTLP (Nm)	155.57(12.39)	172.48(20.94)	10.5	[−2.2;24.7]	1.03 *moderate*	[−0.23;2.29]	88/7/5 *Unclear*
PTRP (Nm)	155.83(11.30)	174.52(17.39)	11.7	[6.4;17.3]	**1.27 *large***	[0.71;1.82]	100/0/0 *Almost certain*
QUADS/HAMS left ratio	62.72(4.69)	67.62(7.25)	7.5	[3.5;11.7]	0.82 *moderate*	[0.39;1.25]	98/2/0 *Very likely*
QUADS/HAMS right ratio	63.53(2.78)	70.58(8.03)	10.6	[1.0;21.1]	**1.94 *large***	[0.19;3.69]	95/2/3 *Likely*

*BM, body mass; FM, fat mass; LM, lean mass; VO_2max_, maximal oxygen consumption; HR_max_, maximal heart rate; PTLQ, peak torque left anterior (quadriceps); PTRQ, peak torque right anterior (quadriceps); PTLP, peak torque left posterior (hamstrings); PTRP, peak torque right posterior (hamstrings); QUADS/HAMS, quadriceps/hamstrings. Bold values: large effect size.*

**FIGURE 2:**
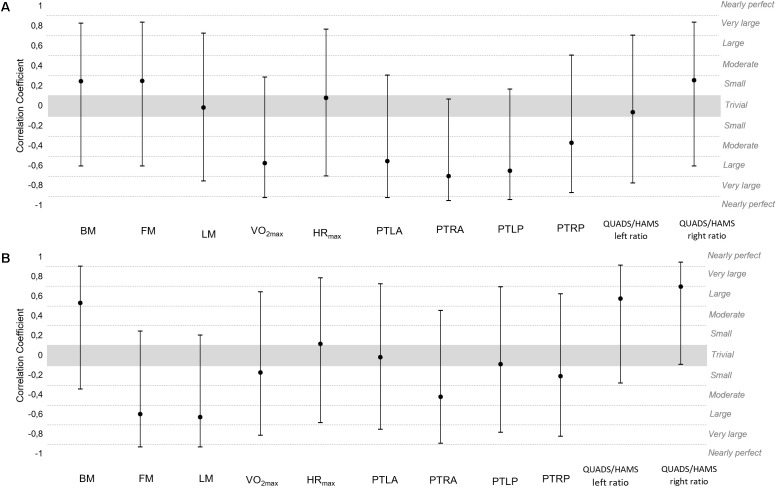
Correlation coefficients (90%CI) of **(A)** sum of duration and **(B)** sum of total distance with % of differences (pre-post) of body composition and fitness variables.

Results from testing associations between the sum of load variables during the 10-week period and percentage of differences (pre-post) in body composition and fitness variables can be observed in Figure 2, 3. Large inverse correlations were found between the sum of training duration and % difference in VO_2max_ [−0.58, (−0.92;0.29)], PTLA [−0.56, (−0.92;0.31)] and PTLP [−0.65, (−0.94;0.17)]. Very large inverse correlations were found between the sum of training duration and % difference in PTRA [−0.70, (−0.95;0.07)].

**FIGURE 3:**
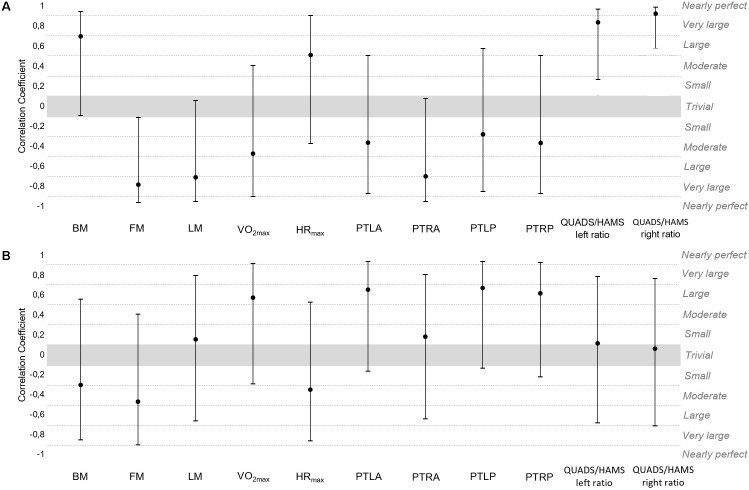
Correlation coefficients (90%CI) of **(A)** sum of sprinting distance and **(B)** acceleration sum with % of differences (pre-post) of body composition and fitness variables. BM, body mass; FM, fat mass; LM, lean mass; VO_2max_, maximal oxygen consumption; HR_max_, maximal heart rate; PTLQ, peak torque left anterior (quadriceps); PTRQ, peak torque right anterior (quadriceps); PTLP, peak torque left posterior (hamstrings); PTRP, peak torque right posterior (hamstrings); QUADS/HAMS, quadriceps/hamstrings.

Large positive correlations were found between the sum of total distance and % difference in BM [0.53, (−0.34;0.91)] and QUADS/HAMS left ratio [0.58, (−0.28;0.92)]. Very large positive correlations were found between the sum of total distance and % differences in QUADS/HAMS right ratio [0.70, (−0.09;0.95)]. Large negative correlations were found between the sum of total distance and % difference in FM [−0.60, (−0.93;0.25)] and LM [−0.63, (−0.93;0.21)].

Large positive correlations were found between the sum of sprinting distance and % difference in BM [0.70, (−0.09;0.95)], HR_max_ [0.51, (−0.37;0.91)], QUADS/HAMS left ratio [0.84, (0.27;0.97)] and QUADS/HAMS right ratio [0.92, (0.58;0.99)]. Very large negative correlations were found between the sum of sprinting distance and % difference in FM [−0.79, (−0.96;-0.11)], LM [−0.71, (−0.95;0.06)] and PTRA [−0.70, (−0.95;0.08)].

Large positive correlations were found between sum of acceleration sum and % difference in VO_2max_ [0.58, (−0.29;0.92)], PTLA [0.66, (−0.16;0.94)], PTLP [0.68, (−0.13;0.94)] and PTRP [0.62, (−0.22;0.93)].

## Discussion

The dose-response relationship was the subject of this research, and was observed in professional soccer players during a 10-week period. The first analysis revealed that body composition and fitness variables changed over the testing period. Specifically, LM likely decreased to a large extent from the first assessment to the second; VO_2max_ almost certainly increased to a large extent; moderate-to-large increases in hamstrings strength were verified, and agonist/antagonist ratios likely increased to a large extent (mainly in the right leg). During the study, players were exposed to 5383 min of training, covered 371.22 km, sprinted almost 9 km, and had an external acceleration sum of 20807 arbitrary units.

Body composition changes during the season have been reported in some studies, showing that these changes can be highly dependent on factors such as training intensity, exposure to match time, or diet (Caldwell and Peters, 2009; Milanese et al., 2015). Generally, FM is the most common indicator analyzed. In some studies it were reported fluctuations with decreases in FM during the first half of the season (Caldwell and Peters, 2009; Devlin et al., 2017); however, such a tendency was not confirmed in other studies (Reinke et al., 2009; Bonuccelli et al., 2012; D’Ascenzi et al., 2015). In our study, possible increases in FM were observed to a small extent (2.9%) and likely decreases in LM were reported to a large extent (−2.2%). The decrease of LM should be carefully interpreted because we have collected the overall LM and not tested the variations in body regions. Such limitation could be an explanation for this result and should be highlighted as a study limitation. In fact, fluctuations of fat and LM are not in line with the findings of previous studies (Carling and Orhant, 2010; Meckel et al., 2018) which have suggested decreases in FM occur during the first months of the season. However, such studies (Carling and Orhant, 2010; Meckel et al., 2018) did not report possible variables to explain the response (namely, the training dose). In our case, the accumulated sprinting distance was largely and negatively associated with changes in fat and LM. In fact, sprinting distance was the variable with the strongest correlation with body composition factors, suggesting that high-intensity distances covered by players may lead to negative associations with fat and LM. This evidence is surprising, considering that greater FM may be associated with decremental effect on sporting performance. However, changes in body composition may occur by complementary processes that justify the findings. Future studies should consider reanalyzing this situation and, in particular, controlling the dietary and supplementary intake. However, despite the magnitudes of the correlations found in this study, the confidence of interval of correlations overtakes the positive threshold. As such, the results are unclear in terms of inference.

Almost certain large increases in VO_2max_ (7.5%) were observed in the period of analysis. Possibly, this improvement can be attributed to the continuous training in moderate-to-vigorous activities that are closely related to improving the aerobic capacity. Despite not using the same measuring criteria, studies that tracked VO_2max_ fluctuations throughout a season have shown improvements in aerobic capacity (Kalapotharakos et al., 2011; Castagna et al., 2013). This physiological variable seems to be sensitive to the dose imposed by intense training; this is based on the fact that the highest magnitude of correlations was found within the acceleration sum, which represents the overall accelerations on the three axes of the tracker devices. In this regard, a systematic and meta-analytic analysis in soccer and other team football codes has shown possibly very large and large associations between external (i.e., acceleration load) and internal measures (e.g., training impulse and session-RPE) (McLaren et al., 2018). This may explain why players with increases in higher accelerations sum showed higher improvements in VO_2max_. In that sense, a possibly great density of actions may be linked with the saturation of cardiovascular system, representing an accumulated stimulus during the testing period which resulted in adaptations of the aerobic capacity. A previous study conducted among elite soccer players revealed that, despite the overall time of training sessions, only the high-intensity time spent above 90% of HR_max_ was related to changes in aerobic fitness (Castagna et al., 2011). However, some cautions should be made in the interpretations mainly because the VO_2max_ may be governed by many other factors such as the economy of running and the velocity/power output at which players normally train (Borresen and Lambert, 2009).

Improvements in isokinetic strength variables were observed during the period of analysis. Peak torque flexion was moderately-to-largely greater in the second assessment, and QUADS/HAMS ratios also moderately-to-largely increased. These results are in line with those of a previous study conducted on professional soccer players that revealed positive changes in peak torque flexion and QUADS/HAMS ratios throughout the season, although significant differences were not found (Eniseler et al., 2012). The correlations revealed that accumulated total distances, sprinting distances, and accelerations sums largely contributed to positive increases in the strength levels of players (flexion and QUADS/HAMS ratios). Possibly, the high-speed intensity (mainly sprinting) represented a contractible enough stimulus (in eccentric phase) to the players that contributed to beneficial adaptations in the isokinetic strength. In fact, some findings have been suggesting that the accumulated accelerations/decelerations load and very high-speed running (>19.8 km/h) is related with an increased risk of a non-contact injury (e.g., muscle injury such as hamstring strains). This may be caused by the fact that these specific metrics were clearly associated with the fatigue and mechanical stress experienced by players (Jones et al., 2016; Gabbett, 2018). It is well accepted that – when not proper managed – it may result in an injury. Oppositely, when training and recovery are properly managed, it may lead to an increase in mechanical capacity (e.g., tissue aggression leads to tissue adaptation) (Malone et al., 2017; Gabbett, 2018). However, we have not analyzed the complementary training of the players (e.g., strength and conditioning sessions) and this represents a limitation of our study. Nevertheless, it was ensured that only one endurance strength session was performed by the players, and so little stimulus alone may not be enough to justify the improvements in isokinetic strength.

Notwithstanding the interesting evidence revealed in our study, there were some limitations. Sprinting speed was not individualized, and thus, the threshold was the same for all players. An individualized threshold would probably help to detect better associations with changes in fitness variables. Moreover, the internal load was not considered in our study. Variables such as VO_2max_ or body composition would certainly add some interesting information about the physiological impact of training sessions. The type of exercises and training content were not analyzed either, and would be important in future studies in describing the time dedicated to each type of exercise in order to better identify the effects on the dose-response analysis. Complementary training was not measured; however, it was ensured that all the players performed only one strength training exercise during the week. Future studies should include the load during complementary training. Another limitation was that the isokinetic strength was only tested at 60°/s. This was justified by the really tight schedule of collecting data with professional players. The lowest speed was chosen based on the lowest risk for players in the beginning of the season. Despite these limitations, the present study was one of the first that tested the dose-response in terms of body composition variables and isokinetic strength in professional soccer players. Moreover, the large associations between external load variables and changes in fitness parameters revealed that training load monitoring can be used to properly identify the effects of training on players and to optimize the planning to the player’s needs. A more individualized approach regarding the load imposed and a careful and regular assessment to the body composition and fitness variables throughout the season may help coaches to ensure the players’ highest level of readiness so that they can perform better.

## Conclusion

This study analyzed the associations between accumulated external load variables and changes in body composition and fitness variables after a 10-week period of soccer training. Main findings regarding the pre- and post-assessments revealed almost certain large increases of VO_2max_ (7.5%), moderate-to-large increases in hamstrings peak torque and likely moderate-to-large increases of agonist/antagonist ratios of isokinetic strength. Associations with load variables revealed that the accumulated duration of sessions was largely and inversely associated with % of changes in VO_2max_ and hamstrings and quadriceps peak torque, possibly suggesting that it is not the amount but the quality/intensity of the training that determines the improvements. Accumulated total distances were largely and positively correlated with BM and QUADS/HAMS ratio changes, thus more distance may represent more possibility to improve in those variables. Accumulated sprinting distances were largely and positively associated with changes in BM, HR_max_, and QUADS/HAMS ratios, suggesting that more sprinting may contribute to improving the variables. In conclusion, we suggest that higher values of accumulated sprinting distances may benefit QUADS/HAMS strength ratios and that accumulated accelerations may be associated closely with benefits in aerobic capacity as analyzed by VO_2max_.

## Author Contributions

FC conceived the study. FC, PN, TR, and BK designed the study. FC collected data. FC analyzed and interpreted the data and drafted the manuscript. FC, PN, TR, and BK revised the manuscript and approved the final version.

## Conflict of Interest Statement

The authors declare that the research was conducted in the absence of any commercial or financial relationships that could be construed as a potential conflict of interest.
